# Management and domestication of cattle (*Bos taurus*) in Neolithic Southwest Asia

**DOI:** 10.1093/af/vfab015

**Published:** 2021-06-19

**Authors:** Benjamin S Arbuckle, Theo M Kassebaum

**Affiliations:** Department of Anthropology, University of North Carolina at Chapel Hill, Chapel Hill, NC 27599, USA

**Keywords:** animal management, aurochs, cattle, domestication, Neolithic Southwest Asia

**Implications**

The traditional narrative that taurine cattle domestication occurred 8500 BC in the Euphrates valley, Syria is critiqued.
Domestic cattle are argued to appear later than widely acknowledged in a wide area of Southwest Asia.
The “pre-domestic management” of cattle preceded the appearance of a domestic phenotype perhaps prior to 8500 BC.
Pre-domestic cattle management as well as early morphologically domestic cattle likely emerged in multiple regions of Southwest Asia rather than in one center.

## Introduction

Cattle are one of the most significant animal partners in human history, and the origins of cattle management as well as domestic cattle have been the focus of scholarly interest for decades (Peake and Fleure, 1927; Reed, 1960). Here, we assess evidence for the management and domestication of taurine cattle (*Bos taurus* Linnaeus 1758) in prehistoric Southwest (**SW**) Asia focusing on archaeological and ancient DNA datasets. Although related, the histories of “cattle management” and “domestic cattle” represent two separate questions. The former refers to a range of techniques including penning, foddering, dairying, mate selection, and selective culling which may vary in intensity, whereas the latter describes biological changes associated with human husbandry, reproductive isolation from progenitors, and selection pressures within an anthropogenic environment (e.g., Dyson, 1953).

**Figure 1.**
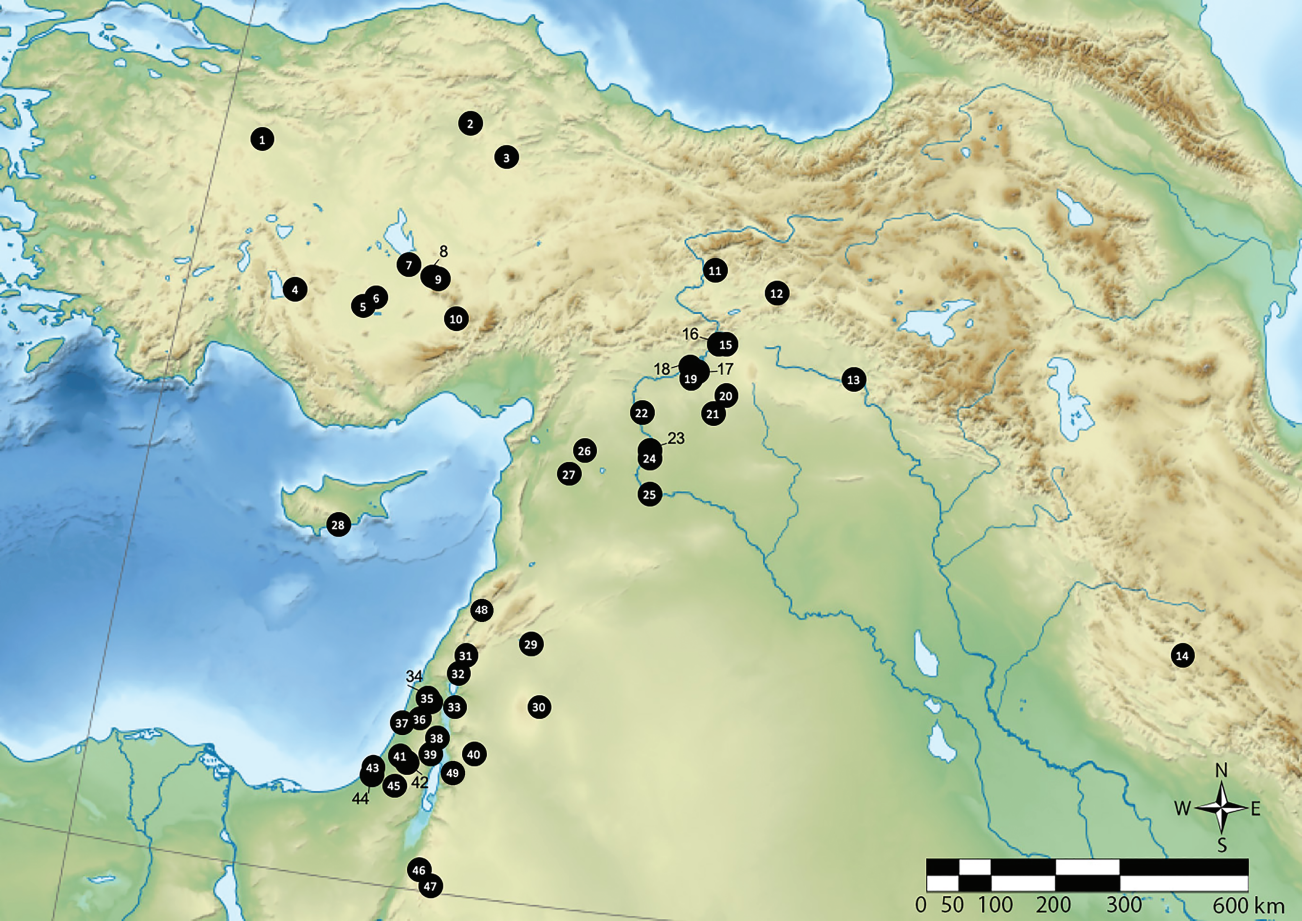
Map showing the location of Neolithic sites mentioned in the text. 1. Orman Fidanlığı, 2. Hattuşa, 3. Çadır Höyük, 4. Erbaba, 5. Çatalhöyük, 6. Boncuklu, 7. Acemhöyük, 8. Musular, 9. Aşıklı Höyük, 10. Köşk Höyük, 11. Cafer Höyük, 12. Çayönü Tepesi, 13. Körtik Tepe, 14. Ganj Dareh, 15. Çavi Tarlaşı, 16. Hassek, 17. Nevalı Çori, 18. Gritille, 19. Lidar Höyük, 20. Göbekli Tepe, 21. Gürcütepe II, 22. Mezraa-Teleilat, 23. Tell Halula, 24. Jerf el-Ahmar, 25. Mureybet, 26. Tell Qaramel, 27. Djade al-Mughara, 28. Shillourokambos, 29. Tell Aswad, 30. Qarassa 3, 31. Hagoshrim, 32. Beisamoun, 33. Sha’ar Hagolan, 34. Kfar HaHoresh, 35. Yiftahel, 36. Mishmar Ha-Emeq, 37. Kebara, 38. Gilgal, 39. Jericho, 40. Ain Ghazal, 41. Abu Gosh, 42. Motza, 43. Afridar, 44. Ashkelon, 45. Grar, 46. Beidha, 47. Basta, 48. Ksar Akil, 49. Teleilat Ghassul.

The dominant narrative describing cattle domestication places its origin within the early farming settlements of the Fertile Crescent region of SW Asia dating to the ninth millennium BC (a period known as the Early Pre-Pottery Neolithic B [**PPNB**]) (Figure 1; Table 1). However, we argue that this narrative is based on models which imagine a single geographic center of innovation and emphasize biometric evidence for body size diminution, i.e., the history of “domestic cattle” rather than “cattle management.” We critique this narrative arguing that the appearance of “domestic cattle” in the ninth millennium BC is largely a mirage and that domestic phenotypes in fact appear in the eighth millennium BC. However, the management of cattle must have preceded changes in phenotype and likely emerged a millennium or more earlier across a wide geographic region including much of the northern and southern Levant—temporal and geographic patterns that fit with recent interpretations of the histories of other livestock species (e.g., Zeder and Hesse, 2000; Martin and Edwards, 2013).

**Table 1.** Chronological terminology and approximate dates in calibrated years BC

Archaeological period	Calibrated years BC
PN	6800/6300–6000/5500
FPPNB/PPNC	7000–6300
LPPNB	7500–7000
MPPNB	8000–7500
EPPNB	8500–8000
PPNA	9500–8500
Late Epipaleolithic	13000–9500

## Defining Domestic Cattle

Domestic cattle (*B. taurus* Linnaeus 1758) are thought to derive from the extinct aurochs (*Bos primigenius* Bojanus 1827), subspecies of which inhabited a wide range of habitats across Eurasia and North Africa (Zeuner, 1963). Recent genomic research has identified two lineages of domestic cattle: the first represented by taurine cattle whose ancestry is thought to lie primarily in Neolithic SW Asia, and the second by zebu cattle (*Bos indicus*, Linnaeus 1758), which can be traced back to a South Asian population of aurochsen (Verdugo et al., 2019). Here, we focus on the early history of taurine cattle in SW Asia, although it is important to note that by the Bronze Age (c. 2000 BC), taurine and zebu cattle became increasingly hybridized, a situation reflected in many modern cattle populations (Verdugo et al., 2019).

Traditional models for identifying the process of cattle domestication focus on identifying changes in phenotype, especially reduced body size and smaller and more variably shaped horns. These changes in phenotype are part of the “domestication syndrome” and have been defined and used by generations of archaeozoologist to distinguish (small) domestic cattle from (large) wild aurochs at prehistoric sites (Duerst, 1908:360). Demographic data relating to age at death and adult sex ratios have also been used to assess cattle domestication as have analyses of stable isotopes from bovid teeth exploring changes in diet and weaning associated with cattle husbandry (e.g., Balasse et al., 1997). Ancient DNA studies have also added to our understanding of the histories of cattle providing evidence for admixture between wild and domestic populations (Verdugo et al., 2019).

## The Traditional Narrative

The upper Euphrates valley of northern Syria has been presented as the “hearth” of taurine cattle domestication. This process is dated to the mid ninth millennium BC associated with the early farming villages of the PPNB (Table 1) (Helmer et al., 2005; Peters et al., 2005). Here, scholars have uncovered a long tradition of *Bos* exploitation among early sedentary communities in a region where the river valley and adjacent grasslands must have supported a large endemic aurochs population (Figure 1).

The earliest of these sites, Tell Mureybet, provides a sequence of occupation from the late Epipaleolithic to the middle PPNB (**MPPNB**) (Table 1). Helmer and Gourichon (2008) argue that Mureybet’s hunters targeted herds of female and juvenile aurochs in the early levels with changes in hunting directed towards increasing production for a growing human population at this large site as well as increased interest in symbolically potent bull aurochs—the remains of which are found in settlements across the region (Cauvin, 1994; Helmer et al., 2004).

Dramatic changes in human cattle relationships are reflected at the site of Dja’de al-Mughara dating to the early PPNB (**EPPNB**), where Helmer argues morphologically domestic cattle are evident for the first time (Helmer et al., 2005). This is based primarily on a small reduction in the size of “male” cattle as evidenced through mixture analysis of a limited set of measurements. The disruption of “natural” sexual dimorphism is interpreted as the result of human management, particularly selection for nonaggressive males, and is seen as the first step in the morphological divergence of domestic cattle. Although presented cautiously by the authors, this small shift in the biometric properties of “male” *Bos* forelimbs is widely reported in the secondary literature as the origins of domestic cattle. As a result, “8500 BC, northern Syria” is the answer that will likely be reported if one queries “when were cattle domesticated?” in an online search engine.

Following the appearance of “domestic” cattle in the Euphrates valley, they are reported on the island of Cyprus at the end of the ninth millennium BC (Vigne, 2011b:1072). Scholars have further traced the spread of a domestic cattle phenotype into neighboring regions including Anatolia and SE Europe, the southern Levant, North Africa, Iran, and the Caucasus in a time transgressive pattern. It is frequently stated that cattle husbandry spread slowly within SW Asia only appearing in the mid seventh millennium BC in central Anatolia and as late as the sixth millennium BC in the southern Levant and Zagros regions (Arbuckle, 2013; Marom and Bar-Oz, 2013; Arbuckle et al., 2016).

This narrative has been incorporated into ancient DNA studies giving it further credence and legitimacy. In a widely cited paper, Bollongino argues that a combination of ancient and modern mitochondrial DNA sequences suggests that as few as 80 female aurochs could have initially been involved in the domestication process which is seen as a geographically and temporally “limited phenomenon” centered in one or two Neolithic villages, such as Dja’de (Bollongino et al., 2012:2103). However, Verdugo et al. (2019) emphasize that later admixtures have fundamentally hidden the early genetic history of cattle including extensive hybridization with zebu cattle from South Asia. Verdugo’s analysis perpetuates other aspects of the traditional cattle domestication narrative; however, including the notion that domestic taurine cattle are derived from a “restricted northern Fertile Crescent genetic background” and that phenotype (particularly body size) can be used to distinguish domestic cattle from aurochs (Verdugo et al., 2019:175).

## Origins of Domestic Cattle

Despite the success of the Euphrates-EPPNB cattle domestication narrative, we argue that the history of cattle domestication is more complex. Zooarchaeological evidence from the Tigris drainage in southeastern Turkey is particularly important showing an alternative history of cattle domestication in a neighboring region. Here, the site of Çayönü Tepesi provides a time sequence recording changes in *Bos* populations and exploitation from the PPNA through the Pottery Neolithic (**PN**) (Öksüz, 2000; Hongo et al., 2009).

At Çayönü, *Bos* remains are abundant in the earliest levels (PPNA) representing c. 20% of the mammalian remains and they exhibit large body size and a sex distribution reflecting the targeting of female aurochsen—similar to the situation documented in the early layers of Mureybet. This pattern of exploiting morphologically wild females continues into the EPPNB and *Bos* remains increase dramatically in the subsequent MPPNB where smaller “domestic” individuals appear for the first time (Hongo et al., 2009). In the following phase, dated to c. 7500 BC, cattle reach their maximum abundance at Çayönü, but are phenotypically identical to the aurochs of the earlier PPNA period. A “permanent” decrease in size is only evident around 7000 BC (late PPNB [**LPPNB**]) followed by continued decrease in cattle size into the PN (Hongo et al., 2009, figure 1). Moreover, age at death data show wide variability through time but with a notable increase in the culling of juveniles in the LPPNB and PN. Finally, shifts in both C and N isotopes from cattle teeth are evident in the early MPPNB, suggesting changes in *Bos* diets beginning in the late ninth millennium BC and becoming more apparent in the LPPNB. This combination of datasets presents a complicated picture which is difficult to fit into a simple linear narrative (although see Peters et al., 2017).

In central Anatolia, faunal evidence for *Bos* exploitation reflects yet another pattern. Although Perkins (1969) argued for early cattle domestication at the Neolithic village of Çatalhöyük, subsequent faunal work has described a tradition of aurochs hunting which targeted adult animals and large males, elements of which were sometimes curated within houses (Baird et al., 2018). This focus on large, adult bulls is also evident in the nearby uplands of Cappadocia in the eighth millennium BC  (Russell et al., 2005). At Çatalhöyük, phenotypically domestic cattle are documented in the mid seventh millennium BC reflecting a curious “delay” in the appearance of domestic cattle in a region with a long tradition of sedentary farming, intensive *Bos* exploitation, and contact with cattle herding neighbors (Arbuckle, 2013; Russell et al., 2013). Peters et al. (2013, 2017) have hypothesized that prior to the appearance of domesticates, morphologically wild *Bos* populations at Çatalhöyük may have been under human management with herders intentionally maintaining a wild phenotype through regular introgression with bull aurochs.

In the southern Levant, the traditional narrative argues that domestic cattle were a late addition to the animal economies of the region (Horwitz et al., 1999). It is frequently reported that “full domestication” of cattle occurred in the sixth millennium BC (PN) (Marom and Bar-Oz, 2013). However, at Tell Aswad in the Damascus basin, changes in horn morphology and a loss of sexual dimorphism in the MPPNB suggest that “domestic” cattle were present prior to the PN in the southern Levant (Helmer and Gourichon, 2008). Helmer and Gourichon (2008:138) also note the presence of pathologies thought to represent the use of cattle for labor and hypothesize that milk was also exploited in the eighth millennium BC (Helmer et al., 2018). In addition, small-sized “domestic” cattle have been identified at Yiftahel in Israel, and Basta and Ain Ghazal in Jordan dating to the eighth millennium BC (Hecker, 1975; von den Driesch and Wodtke, 1997; Becker, 2002; Sapir-Hen et al., 2016). In their summary of cattle domestication in the southern Levant, Horwitz and Ducos (2005:219) state that cattle “clearly exhibit the morphological and metrical changes associated with domestication” in the eighth millennium BC, and Munro et al. (2018) have recently argued that shifts towards cattle management began as early as the ninth millennium (EPPNB) in the southern Levant, completely erasing the perceived time lag with the Euphrates valley.

Analysis of genetic evidence from ancient cattle in SW Asia raises further questions about the notion of a single center for cattle domestication. Verdugo’s important analysis of genomes from 67 ancient bovines shows three divergent Neolithic lineages in SW Asia (Verdugo et al., 2019). Among these, “A” is reflected in the early Neolithic Balkans (but with its origins somewhere in SW Asia); “B” is identified in Neolithic Anatolia and Iran; and “C” is found in the southern Levant. These genetic results suggest that multiple regional populations of aurochsen were incorporated into Neolithic herds, especially north and south of the Taurus, raising questions about the need to center cattle domestication on the northern Fertile Crescent.

## Origins of *Bos* Management

The dominant view of cattle domestication processes focuses on a single center in the Euphrates basin and the emergence of “domestic” forms of cattle in the EPPNB despite questions about the scale of phenotypic changes at this time and evidence for early cattle management in other regions of SW Asia. In contrast, the idea that animal management preceded morphological changes and was geographically widespread has been at the core of work exploring the origins of the management of other livestock taxa for decades. These ideas are relevant for our understanding of the history of cattle as well.

Intensive regimes of “pre-domestic” animal management (i.e., management without clear morphological changes) have been documented for livestock progenitor species across the Fertile Crescent region in the early Holocene. At Ganj Dareh in Iran and Aşıklı Höyük in central Anatolia, evidence for selective culling patterns, foddering, and onsite penning and use of animal dung indicates that morphologically wild sheep and goats were intensely managed in the ninth millennium BC (Zeder and Hesse, 2000; Stiner et al., 2014). These practices persisted for centuries and are not isolated. Similar arguments for the early management of morphologically wild ungulates have been made across SW Asia (e.g., Hecker, 1975; Horwitz, 2003; Vigne, 2011a). These management strategies predating morphological changes have been variously described by scholars as incipient domestication, cultural control, proto-elévage, proto-domestication, and pre-domestic management (Hecker, 1975; Vigne et al., 2000; Munro et al., 2018).

Models of pre-domestic animal management are therefore not new and have even been previously applied to cattle. At Tell Mureybet, Ducos (1978) described evidence for management but no reduction in body size in the EPPNB occupation as a system of *proto-elévage* reflecting the husbandry of morphologically wild animals. Scholars working in central Anatolia, the Euphrates basin, the southern Levant, and Cyprus have all suggested that morphologically wild cattle were managed for centuries prior to the appearance of morphological features of the domestic syndrome (Ducos, 1978; Monahan, 2000; Sana and Tornero, 2013; Munro et al., 2018). The hypothesis of local cattle domestication in regions outside of the Euphrates, including Jordan, the Upper Tigris, and North Africa, has also been explored (Becker, 2002; Marshall and Hildebrand, 2002; Munro et al., 2018).

Thus, an alternate model of pre-domestic cattle management, not limited to a single geographic center and not tied to changes in phenotype, has been available for decades. We argue that this model fits the zooarchaeological and genetic data well. Moreover, analysis of ancient genomes for goats, pigs, and cattle suggests that wild populations from multiple regions of SW Asia contributed to domestic herds reflecting a geographically de-centered domestication process. This is more in line with recent views of the domestication process which tend to emphasize its centerlessness and mosaic nature (Goring-Morris and Belfer-Cohen, 2011) as well as the decoupling of morphological changes from management (Zeder and Hesse, 2000).

One of the problems associated with identifying the appearance of domestic cattle is confusion regarding “how small is small enough” to be considered “domestic”? Although Helmer et al. (2005:90) argue that the individuals from EPPNB Dja’de and MPPNB Halula “are clearly smaller” than the aurochs from earlier sites, the decreases in mean size are very small (Helmer et al., 2005: table 1); a similar situation is evident for the cattle from Shillourokambos (Vigne, 2011b:1070). Moreover, for Saña and Tornero (2013:291), “clearly domestic” (i.e., small sized) cattle are only present at Halula in the early PN (c. seventh millennium BC) rather than the MPPNB. Similar arguments have been echoed at Mezraa-Teleilat (Ilgezdi, 2008) and Gritille (Monahan, 2000) on the Turkish Euphrates and are also widely expressed in the southern Levant (Marom and Bar-Oz, 2013) perhaps reflecting different expectations in regards to the scale of size diminution associated with domestication.

**Figure 2.**
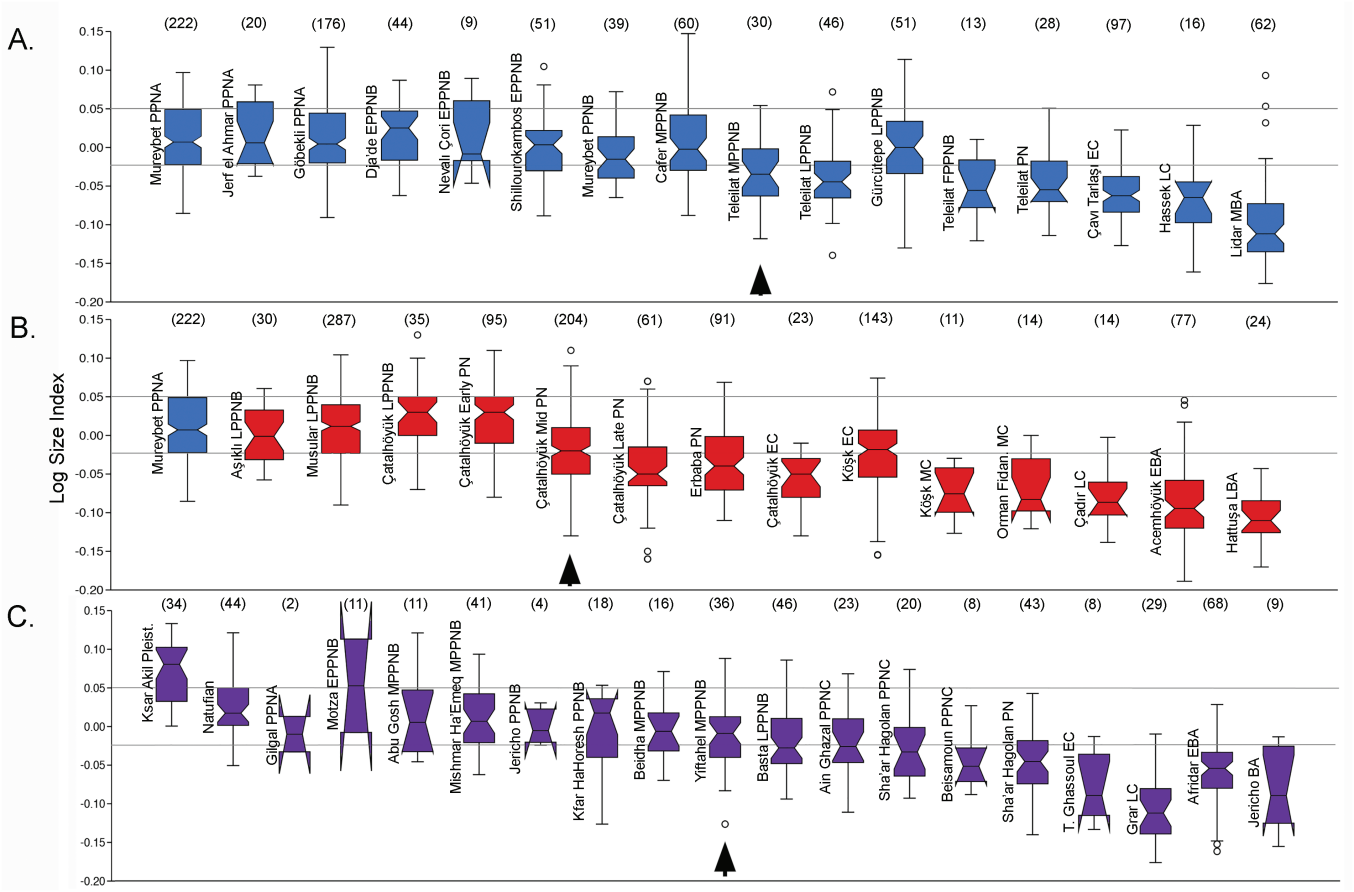
Summaries of biometric data for prehistoric *Bos* from sites in SW Asia presented using LSI. Boxplots of LSI data for selected sites from (A) the Euphrates valley (including Shillourokambos, Cyprus); (B) central Anatolia; and (C) the southern Levant. Horizontal lines represent the interquartile range for LSI measurements from Mureybet (I–III). Arrows indicate earliest significant decrease in size (analysis of variance Tukeys pairwise test *P* < 0.05). EBA, Early Bronze Age; EC, Early Chalcolithic; LBA, Late Bronze Age; LC, Late Chalcolithic; MBA, Middle Bronze Age; MC, Middle Chalcolithic. Sample size in parentheses. See Supplementary Material for data references.

**Figure 3.**
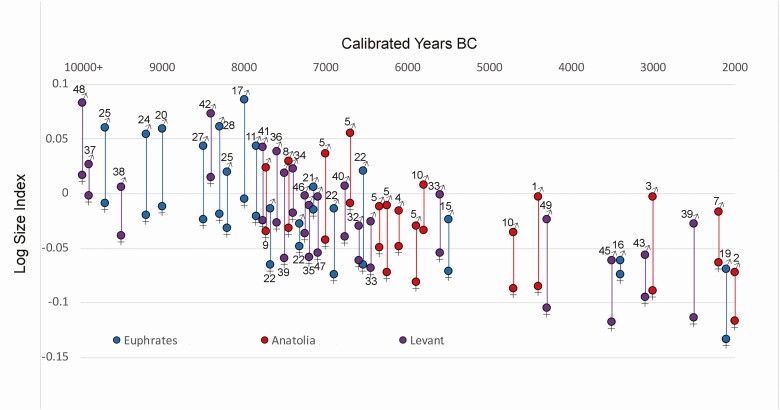
Mean LSI values for both “male” (♂) and “female” (♀) *Bos* from the Euphrates valley (blue) (Shillourokambos, Cyprus included), central Anatolia (red), and the southern Levant (purple) based on mixture analysis (Past v3.20). Sites labeled as in Figure 1.

However, when we look at summaries of biometric data representing long time sequences in the Euphrates basin, central Anatolia, and the southern Levant, we can see broad patterns of change over time within their regional context (Figure 2). Biometric data are presented using the Log Size Index (**LSI**) which compares archaeological measurements against those of a standard animal—in this case, a cow aurochs from the Mesolithic site of Ullerslev, Denmark (Meadow, 1999; Steppan, 2001). Values above “0” reflect dimensions larger than those of the standard while negative values are smaller. In Figure 3, we have generated mean values for “male” and “female” *Bos* using mixture analysis in order to further assess the nature of size change in this sexually dimorphic species.

In Figure 2, we use the interquartile range of the LSI values for aurochs from Mureybet (I–III) to model the size parameters for a SW Asian aurochs population. For the Euphrates region, the earliest large-scale reduction in size, evident as the LSI median moves below the interquartile range of the Mureybet aurochs, is at MPPNB Mezraa-Teleilat on the Turkish Euphrates dating to the early-mid eighth millennium BC (Figure 2A). Despite arguments that early “domestic” cattle phenotypes were established in the EPPNB at Dja’de and Shillourokambos, Cyprus, these populations are broadly similar to the morphologically wild cattle from PPNA Mureybet, Jerf el-Ahmar, Göbekli Tepe, and the Natufian southern Levant (also see Figure 3). Body size continues to decline at Mezraa-Teleilat in the LPPNB and into the PN. However, inter-site variability persists with much larger cattle (including both “males” and “females” [Figure 3]) present at PPNB Mureybet and LPPNB Gürcütepe compared to contemporary sites in the Euphrates valley (Figure 2). Rather than reaching a stable “domestic phenotype” in the Neolithic, body size continues to change over time with dramatic declines and wide variability evident in the late Chalcolithic and Bronze Age.

In Central Anatolia, LSI values for *Bos* at eighth millennium Aşıklı and Musular are comparable in size to Euphrates aurochs, and, at Çatalhöyük, the largest “male” and “female” sizes are evident in the early seventh millennium BC (Figures 2B and 3). Although a decline in body size clearly takes place in the mid seventh millennium in this region, variability is again evident with cattle from contemporaneous Early Chalcolithic (**EC**) Çatalhöyük and Köşk Höyük displaying very different LSI profiles and diminution continuing into the Chalcolithic and Bronze Age.

In the southern Levant (Figure 2C), *Bos* remains from Epipaleolithic, PPNA, EPPNB, and MPPNB sites are broadly comparable in size to Euphrates aurochs—with “male” and “female” LSI means from MPPNB Mishmar Ha-Emeq very similar to those from PPNA Jerf el-Ahmar (Figure 3). Notably smaller cattle appear at some MPPNB sites including Yiftahel and Basta, where both “male” and “female” mean values drop (Figure 3). Kfar HaHoresh, a mortuary site in Israel where cattle feature in feasting practices, includes material from the LPPNB which explains the presence of small-sized cattle at this site otherwise dominated by large aurochs-sized *Bos* dating to the EPPNB (Meier et al., 2016). More dramatic declines in size in the southern Levant are evident in the seventh millennium (PPNC) and in the PN, where “male” and “female” means continue to decline (Figure 3). In the Chalcolithic and Bronze Age, size declines precipitously but also exhibits significant heterogeneity.

These broad biometric summaries show three important features of the temporal and geographic patterns of size change in SW Asian *Bos* which add to our understanding of cattle domestication processes. First, the size change argued to represent early domestication in the EPPNB is subtle to the point of being unobservable using LSI transformed measurements. Although Vigne (2011b:1068) notes that size diminution in the EPPNB is only weakly expressed, and primarily as a disruption in sexual dimorphism, this point has largely been lost in the secondary literature where “domestic cattle” are regularly reported as originating in the ninth millennium BC. Second, large-scale decreases in body size are apparent in the Euphrates valley only in the eighth millennium BC when they also begin to appear in the southern Levant. Third, body size continues to change dramatically in later periods emphasizing that managed cattle are characterized by phenotypic variability in all periods.

These biometric patterns indicate that body size is a dynamic variable which has temporal and geographic dimensions not clearly linked to categories of wild versus domestic. In their careful study of the cattle from Tell Aswad, Helmer and Gourichon (2008:136) warn that body size is not a good criterion for distinguishing wild and domestic cattle and that large size does not necessarily equate to a wild animal, thereby recognizing the problems of conflating phenotype with management (also Helmer et al., 2018:85).

From current archaeological data, we are able to answer the question “when do phenotypically domestic cattle appear in the archaeological record”? Significant changes in body size and horn shape are documented in the eighth millennium BC (MPPNB) in the Upper Euphrates valley, the Upper Tigris valley, and in the Damascus basin. This correlates with regionwide increases in caprine pastoralism, agricultural productivity, and inter-regional connectivity (although not homogenization) taking place within the so-called PPNB “interaction sphere” (Arbuckle and Atici 2013; Borrell and Molist 2014). However, if we decouple phenotype from management, we are left with the question “when and where did cattle management emerge”?

Early sedentary food-producing communities of the Fertile Crescent were centers of “experimental” pre-domestic animal management practices at least as early as the ninth millennium BC (Arbuckle and Atici, 2013; Peters et al., 2017; Munro et al., 2018). Moreover, morphologically wild cattle were transported to Cyprus by the end of the ninth millennium BC providing a *terminus ante quem* for pre-domestic cattle management. This leads us to hypothesize that early cattle management was practiced in a variety of forms in early sedentary villages dating to the 10th and early 9th millennia BC (PPNA and EPPNB) and perhaps even extending back into the Younger Dryas (11th millennium BC). Geographically, we hypothesize that diverse, local management traditions emerged in multiple contemporary communities in the upper Euphrates and Tigris valleys, the Jordan Valley, Mediterranean coast, and central Anatolia.

Likely candidates for loci of early management include sites such as Mureybet, Qarassa 3, and Tell Qaramel in Syria, and Göbekli Tepe, Körtik Tepe, and Boncuklu in Anatolia where aurochs remains are abundant (Arbuckle and Özkaya, 2007; Gourichon and Helmer, 2008; Ibañez et al., 2010; Grezak, 2012; Baird et al., 2018). At Mureybet, Ducos (1978) suggested that morphologically wild cattle were under human management in the EPPNB. It seems likely that at least some of the morphologically wild cattle at Mureybet were subject to a suite of management strategies in earlier periods as well. Moreover, at Göbekli Tepe in southeastern Turkey, Peters et al. (2013:97) noted that the demographic profile for *Bos* suggests “deliberate manipulation” of this population in the PPNA, suggesting that pre-domestic management may have been among the exploitation techniques applied to aurochs at this site.

In central Anatolia, it has been argued that aurochs were hunted prior to the appearance of domestic phenotypes in the mid seventh millennium BC (Russell et al., 2005; Arbuckle, 2013; Pawłowska, 2020). However, given the symbolic, social, and economic importance of cattle in the region, it is plausible that aurochs were subject to forms of management including penning, foddering, and selective culling in the earliest levels of Çatalhöyük, and perhaps at earlier sites in the region such as Boncuklu.

If, as we hypothesize, pre-domestic cattle management was practiced in villages of the PPNA and EPPNB across the Fertile Crescent, why do phenotypic changes only become evident in the eighth millennium? We suggest that the answer is related to the nature of pre-domestic management regimes which may have been small in scale, discontinuously applied, and may not have involved the population isolation necessary to accumulate phenotypic changes associated with the domestication syndrome.

As Vigne (2009:157) points out, the earliest management strategies probably included a constellation of techniques representing just a portion of the diverse forms of interaction between humans and aurochs. We expect that the scale of *Bos* management was small at its inception and may have been applied intermittently. It was therefore a complement to, rather than a replacement for, the hunting of aurochs which continued in the region long after the emergence of domestic cattle.

Peters et al. (2015) have argued that the domestication process involved a long period of “learning by doing” involving inevitable failures and initial low success rates—a feature evident in the range of techniques applied to early caprine management and the ultimate failure of cattle management on Neolithic Cyprus (Vigne 2011b; Arbuckle and Atici, 2013; Stiner et al., 2014). Low success rates in raising aurochs in captivity may have necessitated constant restocking from local free-living populations, a feature seen in pre-domestic caprine management which effectively limited the development of domestic phenotypes.

Moreover, it is likely that the goals of early animal management taking place in the context of a hunting economy were not the same as those in later periods. In particular, an emphasis on large males for feasting and display is suggested by demographic profiles at many sites, as well as practices including the caching of cattle remains (especially bucrania) and imagery of bulls, which are evident across SW Asia from the 10th through the early 7th millennia BC (Cauvin, 1994; Helmer et al., 2004). A central goal of pre-domestic herd managers may have been to provide visually impressive animals for socially and cosmologically charged events. These goals may have been met with intermittent bursts of management and frequent recruitment from free ranging populations specifically designed to maintain wild phenotypes.

If cattle were managed in PPNA and EPPNB villages, as we hypothesize, what management techniques were applied to pre-domestic livestock and how do we identify them if they coexisted with hunting techniques? Surprisingly, the practices of early cattle management have not been addressed in recent scholarship but were a lively topic in the past. For example, Peake and Fleur (1927) present a model of incipient cattle domestication in their influential summary of prehistory. The authors suggest the earliest stage of cattle domestication involved the capture, penning, and foddering of a small number of pregnant cow aurochsen (Peake and Fleure, 1927:34). Through a combination of provisioning and familiarization, aurochs cows and their calves became acclimatized to their resource-rich “home.” Allowing cows and calves to graze and return to pens at night would ensure seasonal opportunities to mate with free-living male aurochs. Although more than a century old, this model has the benefit of accommodating the behavioral difficulties of living in close proximity to adult, male aurochsen as well as describing a plausible scenario in which small-scale management regimes which inhibit morphological changes could be applied. Although cattle penning deposits have not been specifically identified in Neolithic SW Asia, it has been suggested based on isotopic evidence that morphologically wild cattle at Kfar HaHoresh were foddered and perhaps penned (Makarewicz et al., 2016).

The symbolic importance of aurochs within PPNA settlements has been widely noted (Cauvin, 1994; Helmer et al., 2004). Although the potent symbolism associated with the largest prey species in SW Asia has been identified as a potential factor in the late domestication of cattle (Vigne, 2009:157; Arbuckle, 2013), the opposite may be true. It may be that the social and cosmological significance of aurochs drove the efforts to capture, pen, and fodder them and also contributed to the slow shift to a domestic phenotype (Peters et al., 2013). The latter may have been intentionally delayed, especially in central Anatolia, where impressive physical appearance seems to have been highly valued.

The types of pre-domestic management strategies hypothesized for the 10th and 9th millennia BC, particularly when situated within a mosaic of other exploitation techniques, pose serious challenges in terms of identification and require a renewed and explicit research focus. Exploring the diets and mobility of individual cattle through isotopic analyses and changes in the skeleton associated with penning may provide indicators of human impact on individual animals (e.g., Zimmermann et al., 2018; Harbers et al., 2020). Studies of ancient cattle genomes may identify the movement of specific lineages such as those brought to Cyprus, further elucidate the origins of domestic cattle within local aurochs populations, or identify phenotypic changes related to human selection such as coat color not evident in the skeletal record. Finally, geomorphological evidence for onsite penning has clarified the early history of sheep and goat management (Stiner et al., 2014; Matthews, 2016; Portillo et al., 2020), and similar evidence for offsite cattle penning may allow us to further tease out the details of evolving human–*Bos* relationships in early sedentary communities.

## Conclusion

The traditional narrative that domestic taurine cattle originated in a few villages in the upper Euphrates valley in northern Syria in the EPPNB is problematic. We argue that this narrative is a mirage based on inconsistent interpretations of biometric evidence for size change and geographically centered models of domestication. Instead, dramatic changes in cattle phenotype including body and horn size are evident in a wide arc including the Upper Euphrates, Upper Tigris, and southern Levant almost a thousand years later (eighth millennium BC).

The breakwater points widely identified as the origins of domestic taurine cattle—the EPPNB in Euphrates and its chronological equivalent on Cyprus, the PPNC or PN in the Jordan Valley, and the PN in central Anatolia—are recognized as important inflection points in human–cattle relationships, notably the widespread appearance of new domestic phenotypes, but they do not represent the beginning of close relationships between humans and aurochs which extend temporally in both directions. Rather, we argue that a long history of pre-domestic cattle management preceded the appearance of “domestic” cattle, whose slow reproductive rates, combined with early herders “learning by doing,” and an apparent preference for the “aurochs aesthetic” likely made it necessary for herders to draw from local aurochs populations thereby inhibiting the appearance of domestic phenotypes.

Instead of focusing on the Euphrates valley in the mid ninth millennium BC, we hypothesize that early cattle management was practiced in many sedentary communities of the PPNA across the Fertile Crescent region. Idiosyncratic and heterogenous systems of *proto-elévage* or pre-domestic management must have emerged in the centuries if not millennia prior to the EPPNB in communities such as Mureybet and Göbekli Tepe as well as contemporary settlements in the Jordan valley and Mediterranean coast where relationships of hunting slowly transformed into management and management, combined with population isolation, eventually transformed aurochs into cattle. Through the concentration of a suite of high-resolution analyses of archaeological and archaeogenetic material in these periods and places, we predict scholars in the next decade will produce a new chapter in the history of taurine cattle extending out of the Euphrates valley, past evidence for size change, and temporally beyond the PPNB.

## Supplementary Material

vfab015_suppl_Supplementary-Material

## About the Authors

Benjamin S. Arbuckle is Associate Professor in the Department of Anthropology at the University of North Carolina at Chapel Hill, USA. He received a PhD in Anthropology from Harvard University with a thesis focused on sheep and goat pastoralism in prehistoric Anatolia. Research interests include the origins of domestic livestock, zooarchaeology, and prehistory of SW Asia especially Turkey.

Theo M. Kassebaum is a doctoral candidate in the Department of Anthropology at the University of North Carolina at Chapel Hill, USA. She received an MSc degree in Zooarchaeology from the University of York, UK and a BA in Anthropology from the University of Chicago. Research interests focus around economic resilience and animal management in times of environmental and political unrest especially in Bronze Age SW Asia.
