# Supplementary material for: Management and domestication of cattle (Bos taurus) in Neolithic Southwest Asia
Source: Anim Front. 2021 Jun 19;11(3):10–9. doi: 10.1093/af/vfab015 (PMC8214434; doi:10.1093/af/vfab015)
Supplement: vfab015_suppl_Supplementary-Material [file vfab015_suppl_supplementary-material.docx]

**Supplement:**

**References for Figures 2 and 3:**

Mureybet ‘PPNA’ includes levels I-III. Ksar Akil data include Paleolithic through Epipaleolithic; ‘Natufian’ data include Ain Mallaha, Ein Gev, Hayonim, El Wad, and Kebara Cave B; Motza data represents mean, maximum and minimum values only. Boxplot labeled Beisamoun PPNC also includes data from Ashkelon.

| Site | Region | Sample size (LSI) | Reference |
| --- | --- | --- | --- |
| Mureybet I-III | Northern Levant | 222 | *Gourichon and Helmer 2008* |
| Jerf el Ahmar | Northern Levant | 20 | *Helmer and Gourichon 2008* |
| Göbekli II/III | Northern Levant | 176 | *Peters et al. 2017* |
| Dja’de al Mughara | Northern Levant | 44 | *Helmer et al. 2005; Helmer and Gourichon 2008* |
| Nevalı Çori | Northern Levant | 9 | *Peters et al. 2017* |
| Shillourokambos | Cyprus | 51 | *Vigne 2011* |
| Mureybet IV | Northern Levant | 39 | *Gourichon and Helmer 2008* |
| Cafer | Northern Levant | 60 | *Helmer 2008* |
| Mezra-Teleilat MPPNB | Northern Levant | 30 | *Ilgezdi 2008* |
| Mezra-Teleilat LPPNB | Northern Levant | 46 | *Ilgezdi 2008* |
| Gürücütepe II | Northern Levant | 51 | *Peters et al. 2013* |
| Mezra-Teleilat FPPNB | Northern Levant | 13 | *Ilgezdi 2008* |
| Mezra-Teleilat PN | Northern Levant | 28 | *Ilgezdi 2008* |
| Çavı Tarlası | Northern Levant | 97 | *Schaffer and Boessneck 1988* |
| Hassek Höyük LC | Northern Levant | 16 | *Boessneck 1992* |
| Lidar Höyük MBA | Northern Levant | 62 | *Kussinger 1988* |
| Aşıklı Höyük II | Anatolia | 30 | *Russell et al. 2005* |
| Musular | Anatolia | 287 | *Russell et al. 2005* |
| Çatalhöyük Aceramic | Anatolia | 35 | *Russell et al. 2013* |
| Çatalhöyük Early PN | Anatolia | 95 | *Russell et al. 2013* |
| Çatalhöyük Mid PN | Anatolia | 204 | *Russell et al. 2013* |
| Çatalhöyük Late PN | Anatolia | 61 | *Marciniak et al. 2013* |
| Erbaba Höyük | Anatolia | 91 | *Arbuckle* *2013a* |
| Çatalhöyük West EC | Anatolia | 23 | *Russell et al. 2013* |
| Köşk Hoyuk EC | Anatolia | 143 | *Arbuckle 2013b* |
| Orman Fidanlığı | Anatolia | 11 | *Uerpmann 2001* |
| Çadır LC | Anatolia | 14 | Author’s data |
| Acemhöyük EBA | Anatolia | 77 | Author’s data |
| Hattuşa LBA | Anatolia | 24 | *Hollenstein and Middea 2014* |
| Ksar Akil | Southern Levant | 34 | *Hooijer 1961* |
| Natufian | Southern Levant | 44 | *Davis 1981; Ducos 1991; Saxon 1974* |
| Gilgal | Southern Levant | 2 | *Ducos 1991; Horwitz et al. 2010* |
| Motza | Southern Levant | 11 | *Sapir-Hen et al. 2016* |
| Abu Gosh | Southern Levant | 11 | *Horwitz 2003* |
| Mishmar Ha-Emeq | Southern Levant | 41 | *Marom and Bar-Oz 2013* |
| Jericho PPNB | Southern Levant | 4 | *Clutton-Brock 1979* |
| Kfar HaHoresh | Southern Levant | 18 | *Meier et al. 2016* |
| Beidha | Southern Levant | 16 | *Hecker 1975* |
| Yiftahel | Southern Levant | 36 | *Sapir-Hen et al. 2016* |
| Basta | Southern Levant | 46 | *Becker 2002* |
| Ain Ghazal PPNC | Southern Levant | 23 | *von den Driesch and Wodtke 1997* |
| Sha’ar Hagolan PPNC | Southern Levant | 20 | *Marom and Bar-Oz 2013* |
| Beisamoun/Ashkelon | Southern Levant | 8 | *Garfinkel et al. 2005; Khalaily et al. 2015* |
| Sha’ar Hagolan PN | Southern Levant | 43 | *Marom and Bar-Oz 2013* |
| Teleilat Ghassoul PN | Southern Levant | 8 | *Davis 1981* |
| Grar LC | Southern Levant | 29 | *Grigson 1995* |
| Afridar EBA | Southern Levant | 68 | *Whitcher 2000* |
| Jericho BA | Southern Levant | 9 | *Clutton-Brock 1979* |

Arbuckle, B.S. 2013a Erbaba Höyük and Suberde Zooarchaeology Data. *Open Context*. DOI: http://dx.doi.org/10.6078/M7C8276W.

2013b Köşk Höyük Faunal Data. *Open Context*. DOI: http://dx.doi.org/10.6078/M7H12ZXT.

Becker, C. 2002. Nothing to do with indigenous domestication? Cattle from Late PPNB Basta*.* In H. Buitenhuis, A. M. Choyke, M. Mashkour, and A. H. Al-Shiyab. (eds), *Archaeozoology of the Near East V. Proceedings of the fifth international symposium on the archaeozoology of southwestern Asia and adjacent areas*. ARC Publication 62. Groningne, The Netherlands: 112-137.

Boessneck, J. 1992. Besprechung der Tierknochen- und Molluskenreste von Hassek Höyük*.* In M. R. Behm-Blancke. (eds), *Hassek Höyük. Naturwissenschaftliche Untersuchungen und lithische Industrie*. Ernst Wasmuth Verlag. Tübingen: 58-74.

Clutton-Brock, Juliet 1979. The mammalian remains from the Jericho Tell. *Proceedings of the Prehistoric Society* *45*:*135-157*.

Davis, Simon JM 1981. The effects of temperature change and domestication on the body size of Late Pleistocene to Holocene mammals of Israel. *Paleobiology*:*101-114*.

Ducos, Pierre 1991. “Bos, Ovis et Capra” dans les sites Néolithiques du Proche Orient. *Paléorient*:*161-168*.

Garfinkel, Yosef, Doron Dag, Brian Hesse, Paula Wapnish, Dolores Rookis, Gideon Hartmann, D. E. Bar-Yosef Mayer, and Omri Lernau 2005. Neolithic Ashkelon: Meat processing and early pastoralism on the Meditarranean coast have it pdf. *Eurasian Prehistory* *3*:*43-72*.

Gourichon, Lionel, and Daniel Helmer 2008. Étude archéozoologique de Mureybet*.* In Juan Jose Ibánez. (eds), *Le site néolithique de Tell Mureybet (Syrie du Nord)*. BAR International Series 1843. Oxford: 115-228.

Grigson, C 1995. Cattle keepers of the northern Negev: animal remains from the Chalcolithic site of Grar*.* In Isaac Gilead. (eds), *Grar: A Chalcolithic site in the northern Negev*. Ben-Gurion University of the Negev Press. Jerusalem: 377-452.

Hecker, H. 1975. The faunal analysis of the primary food animals from the Pre-Pottery Neolithic Beidha (Jordan) PhD Thesis, PhD Dissertation, Columbia University, Ann Arbor, Michigan.

Helmer, D., and L. Gourichon 2008. Premières données sur les modalités de subsistance à Tell Aswad (Syrie, PPNB Moyen et Récent, Néolithique Céramique Ancien) - Fouilles 2001-2005. In E. Vila, L. Gourichon, H. Buitenhuis, and A. Choyke. (eds), *Archaeozoology of the Near East 8*. Maison de l'Orient et de la Méditerranée. Lyon: 119-151.

Helmer, D., L. Gourichon, H. Monchot, J. Peters, and M. Sana Segui 2005. Identifying early domestic cattle from Pre-Pottery Neolithic sites on the Euphrates using sexual dimorphism*.* In J-. D. Vigne, J. Peters, and D. Helmer. (eds), *The first steps of animal domestication: New archaeological approaches. Proceedings of the 9th ICAZ Conference, Durham 2002*. Oxbow. Oxford: 86-95.

Helmer, Daniel 2008. Revision de la faune de Cafer Hoyuk (Malatya, Turquie): apports des methodes de l'analyse des melanges et de l'analyse de Kernel a la mise en evidence de la domestication*.* In Emmanuelle Vila, L. Gourichon, A. Choyke, and H. Buitenhuis. (eds), *Archaeozoology of the Near East VIII*. Maison de l'Orient et de la Mediterranee. Lyon: 169-196.

Hollenstein, Daria, and Geraldine Middea 2014. The faunal remains from the Square Building Horizon in the Valley West of Sarıkale, Bogazköy-Hattusa, Turkey (16th/15th century BC). *Bogazköy-Hattusa Ergebnisse der Ausgrabungen* *24*:*147-215*.

Hooijer, D. A. 1961. *The fossil vertebrates of Ksar Akil*. Brill. Leiden.

Horwitz, L. K. 2003. The Neolithic fauna. In: H. Khalaily and O. Marder, editors, *The*

*Neolithic site of Abu Gosh*. *The 1995 Excavations*. Israeli Antiquities Authority Reports vol 19, Jerusalem. p. 87-101.

Horwitz, Liora Kolska, Tal Simmons, Omri Lernau, and Eitan Tchernov 2010. Fauna from the sites of Gilgal I, II and III*.* In O. Bar-Yosef, A. N. Goring-Morris, and A. Gopher. (eds), *Gilgal: excavations at Early Neolithic sites in the Lower Jordan Valley: the excavations of Tamar Noy*. Oxbow. Oxford: 263-296.

Ilgezdi, Gulçin 2008. The Domestication Process in Southeastern Turkey: The Evidence of Mezraa-Teleilat. *PhD Dissertation, Geowissenschaftlichen Fakultät der Eberhard-Karls-Universität Tübingen*.

Khalaily, HAMOUDI, TALI Kuperman, NIMROD Marom, IANIR Milevski, and DMITRY Yegorov 2015. Beisamun: An Early Pottery Neolithic site in the Hula Basin.‘. *Atiqot* *82*:*1-61*.

Kussinger, Sonja 1988. Tierknochenfunde vom Lidar Höyük (Südostanatolien)*.* PhD, University of Munich, Munich.

Marciniak, Arkadiusz, Kamilla Pawlowska, Nerissa Russell, Katheryn Twiss, Louise Martin, E. Henton, D. C. Orton, Arzu Demirergi, B. Aydinuloglu, R. Daly, C. Christensen, A. L. Atici, Ian Cameron, and Sheelagh Frame 2013 Çatalhöyük Area TP Main Zooarchaeological Dataset. *Open Context*. DOI: http://dx.doi.org/10.6078/M7PK0D3R.

Marom, Nimrod, and Guy Bar-Oz 2013. The prey pathway: A regional history of cattle (Bos taurus) and pig (Sus scrofa) domestication in the northern Jordan Valley, Israel. *PLoS one* *8*:*e55958*.

Meier, Jacqueline S, A Nigel Goring-Morris, and Natalie D Munro 2016. Provisioning the ritual Neolithic site of Kfar HaHoresh, Israel at the dawn of animal management. *PloS one* *11*:*e0166573*.

Peters, Joris, Hijlke Buitenhuis, Gisela Grupe, Klaus Schmidt, and Nadja Pöllath 2013. The long and winding road: ungulate exploitation and domestication in Early Neolithic Anatolia (10000–7000 cal BC)*.* In Sue Colledge, James Conolly, Keith Dobney, Katie Manning, and Stephen Shennan. (eds), *The origins and spread of domestic animals in southwest Asia and Europe*. Left Coast Press. Walnut Creek, CA: 83-114.

Peters, Joris, Nadja Pöllath, and B. S. Arbuckle 2017. The emergence of livestock husbandry in Early Neolithic Anatolia*.* In Umberto Albarella, Hannah Russ, Kim Vickers, and Sarah Viner-Daniels. (eds), *Oxford Handbook of Zooarchaeology*. Oxford University Press. Oxford: 247-265.

Russell, N, K Twiss, S Frame, L Yeomans, L Martin, C Christensen, D Orton, A Demirergi, S Meese, K Pawlowska, B Aydinuloglu, E Henton, A Watson, R Mayon-White, I Cameron, A Erwin, V Dimitrijevic, R Daly, R Symmons, S Yeni, D Carruthers, C Hills, H Buitenhuis, L Leblanc, L Atıcı, D Boric, and A Powell 2013 Çatalhöyük Main Zooarchaeological Dataset. *Open Context*. DOI: http://dx.doi.org/10.6078/M7V985ZW.

Russell, Nerissa, Louise Martin, and Hijlke Buitenhuis 2005. Cattle domestication at Çatalhoyuk revisited. *Current Anthropology* *46 Supplement*:*S101-108*.

Sapir-Hen, Lidar, Tamar Dayan, Hamoudi Khalaily, and Natalie D Munro 2016. Human hunting and nascent animal management at Middle Pre-Pottery Neolithic Yiftah'el, Israel. *PloS one* *11*:*e0156964*.

Saxon, E. C. 1974. The mobile herding economy of Kebarah Cave, Mt Carmel: an economic analysis of the faunal remains. *Journal of Archaeological Science* *1*:*27-45*.

Schäffer, J., and J. Boessneck. 1988. Bericht über die Tierreste aus der halafzeitlichen

Siedlung Çavi Tarlasi (Nisibin, Osttürkei). *Istanbuler Mitteilungen* *38*:*37-62*.

Uerpmann, H.-P. 2001. Remarks on faunal remains from the Chalcolithic sites "Orman Fidanlığı" and "Kes Kaya" near Eskişehir in North-Western Anatolia*.* In Turan Efe. (eds), *The salvage excavations at Orman Fidanlıgı: A Chalcolithic site in inland northwestern Anatolia*. TASK Vakfı Yayınları. Istanbul: 187-210.

Vigne, Jean-Denis 2011. Les bovins (Bos taurus)*.* In Jean Guilaine, François Briois, and Vigne Jean-Denis. (eds), *Shillourokambos: Un établissement néolithique pré-céramique a Chypre. les fouilles du secteur 1*. Editions Errance. Paris: 1059-1073.

von den Driesch, Angela, and Ursula Wodtke 1997. The fauna of 'AIn Ghazal, a major PPN and early PN settlement in central Jordan*.* In H. G. K. Gebel, Z. Kafifi, and G. O. Rollefson. (eds), *The prehistory of Jordan II. Perspectives from 1997. Studies in Early Near Eastern Production, Subsistence and Environment 4*. Ex Oriente. Berlin: 511-556.

Whitcher, Sarah E 2000. Animals, environment and society: a zooarchaeological approach to the Late Chalcolithic-Early Bronze I transition in the southern Levant, University of Edinburgh.
